# Experiences and management of breathing and shortness of breath in patients with chronic heart failure – a qualitative study

**DOI:** 10.1186/s12872-025-05422-z

**Published:** 2025-12-11

**Authors:** Jonna Norman, Åsa Rejnö, Lena Björck

**Affiliations:** 1 Institute of Health and Care Sciences, Sahlgrenska Academy, University of Gothenburg, Gothenburg, Sweden; 2 The Gothenburg University Centre for Person-Centred Care (GPCC), Sahlgrenska Academy, Gothenburg, Sweden; 3 Region Västra Götaland, Sahlgrenska University Hospital, Department of Medicine, Geriatric and Emergency Medicine/Östra, Gothenburg, Sweden; 4 Department of Health Sciences, University West, Trollhättan, Sweden; 5 Skaraborg Institute of Research and Development, Skövde, Sweden; 6 Department of Medicine, Skaraborg Hospital, Skövde, Sweden; 7 Department of Molecular and Clinical Medicine, Sahlgrenska Academy, University of Gothenburg, Gothenburg, Sweden

**Keywords:** Heart failure; qualitative research, Interviews, Symptoms, Dyspnea

## Abstract

**Background:**

Shortness of breath is a cardinal symptom of heart failure. However, little is known about patients’ subjective experiences and how patients with chronic heart failure manage shortness of breath. This study aimed to describe the experiences and management of breathing and shortness of breath in patients with stable symptomatic chronic heart failure.

**Methods:**

This qualitative study used a descriptive design. Data were collected through assessment tool assisted interviews, using the Experiences of Breathing and Shortness of Breath (Exp-BeSoB). Outpatients with stable heart failure in functional classes II − III who were on optimized medical treatment were included. Qualitative content analyses were conducted.

**Results:**

Forty-five participants (median age, 74 years), 37 (82%) reported normal breathing and wellness at rest. Varied personal and multidimensional experiences of shortness of breath were found. Shortness of breath was described as a lack of air and energy and associated with physical exertion and environmental factors. Different, frightening, and challenging shortness of breath experiences also emerged. Participants developed management strategies, that they found effective. Three themes representing patient experience were identified: Shortness of breath *as a life-threatening experience*, *Difficulty breathing slows the body down*,* grabbing one’s focus* and *Breathing is not a problem and of no concern*. Applied personal management strategies differed within and between the themes.

**Conclusions:**

Shortness of breath in stable heart failure can present in various ways and can be effectively managed. Results highlight the importance of clinicians exploring the patients’ breathing symptoms, their thoughts about the symptom, emotional and existential dimensions, impact in daily life, and management strategies, which might help to identify patients’ needs for breathing care. Further research is needed.

## Background

Heart failure is a common and serious condition with severe symptoms that have a negative impact on well-being and survival [1, 2]. The estimated prevalence of chronic heart failure is 2–2,5% and increases to approximately 10% in patients aged >85 years [1]. Shortness of breath, medically known as dyspnea, is a cardinal symptom of heart failure, together with fatigue, and signs of fluid edema, typically in the legs, ankles and/or feet [1, 3–5]. Breathing difficulties are also the most common reasons for hospitalization [6, 7]. Moreover, a worsening of self-reported shortness of breath in those with heart failure is associated with increased mortality [5, 8]. Despite medical treatment and care, breathing symptoms remain problematic [4, 9–12]. Patients are restricted both emotionally and physically, and are unable to live the way they used to [11, 13]. The American Thoracic Society (ATS) define dyspnea as “a subjective experience of breathing discomfort that consists of qualitatively distinct sensations that vary in intensity*”* [3]. In this paper, we use shortness of breath (SOB), synonymous with the medical term “dyspnea”.

Few qualitative studies using explorative interviews have been conducted, and the lived experience and management of breathing and SOB in a stable phase of chronic heart failure is little understood [13–15]. Research has mostly focused on worsening SOB, due to acute heart failure, with decompensation leading to hospitalization [7, 16–20]. Meanwhile, few studies have focused on patients’ experienced severity of breathing symptoms as a primary outcome [8, 21–23]. A common assumption is that SOB is a sensation that varies only in the degree of intensity [21, 23]. However, various qualities of SOB in cardiopulmonary diseases have been identified, such as sensations of the breathing to be heavy or rapid [17, 24]. Patients may also have difficulty in detecting and describing their experiences of being short of breath related to heart failure [16, 18, 25]. Despite the clinical relevance, studies targeting patient experience and management of breathing symptoms in patients with stable chronic heart failure is scarce [4, 26].

Breathing symptoms need to be understood in the context of each patient’s daily life, and the focus should be on self-care to maintain health while living with heart failure [4, 9, 13, 27]. The New York Heart Association (NYHA) is used in clinic to asses symptoms, including SOB, in patients with heart failure [1]. While, the Visual Analog Scale, Numerical Rating Scale, and Likert scales, are used for the self-rating of patients experiencing SOB [8, 9, 21], patients report that it is difficult to respond in meaningful ways on uni-dimensional scales, since it is unclear how or on what basis they should rate their SOB [13]. In accordance with the definition proposed by ATS, experience of SOB derives from interactions among multiple factors [3]. Meanwhile, knowledge about patients’ own experiences and management of breathing while living with chronic heart failure is scarce [13–15, 27]. Broadening the perspective and integrating synthesis of research on other medical conditions with breathing symptoms, such as the theoretical framework on “breathing space”, could be helpful [27].

In clinical encounters, SOB is an important and problematic symptom in outpatients with heart failure that persists despite guideline-specific treatment and care [1, 9]. To the best of our knowledge, few studies have explored breathing, SOB, and the management of breathing in outpatients with stable but still symptomatic chronic heart failure [13–15]. A better understanding of patients’ experiences of breathing and SOB, as well as management strategies, are essential for healthcare professionals to be able to improve care for those with chronic heart failure. Thus, this study aims to describe the experiences and management of breathing and SOB in patients with stable symptomatic chronic heart failure.

## Methods

### Design

This study used a descriptive design with a qualitative approach and was performed in accordance with the Standards for Reporting Qualitative Research checklist [28]. 

The study was conducted as part of a larger project (ClinicalTrial.gov, ID NCT04871178. Registered May 04, 2021. Retrospectively registered). This study was conducted during 2023 to 2024. The present study used the open-ended questions included in an assessment tool in development, Experiences of Breathing and Shortness of Breath (Exp-BeSoB) to explore patient experiences on breathing and SOB at the baseline visit of the feasibility study in the project, which has been described in previous publication [29]. Initially the aim, of the present qualitative study, was to focus only on patients’ experiences with breathing and SOB. Inspired by the work on “breathing space” [27], an expanded perspective on the participants’ experiences in the current study, seemed beneficial. In accordance with this, the aim was expanded to also include how the patients managed SOB. Data were analysed using content analysis [30, 31]. Texts may have multiple meanings, and in this study, we explored the experiences of breathing, SOB and management strategies as expressed by patients with chronic heart failure captured by the open-ended questions and comments on the verbal descriptors, in an assessment tool in development, Exp-BeSoB.

### Assessment tool

The extension of the assessment tool Exp-BeSoB was developed through narration of breathing symptoms in patients with stable chronic heart failure, collected in interviews using the Shortness of Breath in Heart Failure (SOB-HF), in a previous study [32]. The Exp-BeSoB includes verbal descriptors, self-rating scales, and open-ended questions (Table 1). Examples of descriptors on the sensory and physical dimension of SOB were “I’m not getting enough air/oxygen” and “I cannot inhale completely”. In the emotional dimension of SOB, participants were asked to choose from words, such as “irritating”, “sad”, “worrying” etc., which described what they felt when they became short of breath. They were also asked to rate the extent [1–5] to which the descriptor corresponded to what they felt. With the attempt to reduce recall bias of symptoms, the assessment tool included both questions on present and past breathing experiences. The initial open-ended questions were intended to capture personal experiences and management of breathing and shortness of breath in a stable phase of chronic heart failure. While the verbal descriptors were intended to help participants recognise their own experiences in the descriptions of others and add details themselves.

The present study is linked to a broader development process for the Exp-BeSoB, in that it represents one step towards validation of the assessment tool.

### Sample and settings

Fifty patients were enrolled into the project [29] with eligibility criteria: aged ≥ 18 years, diagnosed with stable but symptomatic chronic heart failure in NYHA class II − III and optimized medical treatment [1]. A stable condition was defined as no deterioration within the previous 4 weeks. The adequacy of medical treatment was confirmed by a heart failure specialist. Participants were followed up at an outpatient clinic at a university hospital in western Sweden. Additional inclusion criteria in the present study was a history of SOB experiences within the last 2 years.

### Data collection

Data were collected through assessment tool assisted [33] interviews by the first author (J.N.)

using the Exp-BeSoB in development, an extended version of the SOB-HF [17]. The answers to the open-ended questions (Table 1) and comments on the descriptors were used in this study. The specific research questions were as follows.

- What experiences of shortness of breath do patients with stable heart failure have?
- How is shortness of breath managed?

Forty-three face-to-face interviews were conducted at the outpatient clinic and two at the participants’ homes. The interviews were conducted as a part of a larger project starting in 2012. The first author (J.N.) conducted all the interviews and carefully wrote down verbatim what the participants said in the assessment tool, question by question. Both shorter (if unclear) and longer descriptions were re-read, verified, corrected, or complemented directly by the participants. The narratives were written down together with the participants’ gestures, pauses and emotional reactions.

Written text from each participants’ answers in the Exp-BeSoB, was verbatim transcribed into an audit trail. To verify the accuracy and objectivity of the data transferred to the audit trail, the original handwritten data of all 45 participants, recorded using the Exp-BeSoB assessment tool, were reviewed.

**Table 1:** Open-ended questions used from the assessment tool

The open-ended questions	
How does your breathing feel at this very moment? Where in your body do you feel your breathing?	
Have you experienced shortness of breath in the last 2 years?	No/Yes
If yes, can you describe how it felt then?	
What causes/makes you short of breath? If you become short of breath when you exert yourself, have you exerted yourself to that level in the last week? If no, please add a comment to your answer. If yes, please add a comment to your answer.	No/Yes
How do you deal with your shortness of breath? How long are you short of breath each time?	
What are your thoughts on your breathing and shortness of breath?	

### Data analysis

The data were analysed manually, without use of any software, in several phases using qualitative content analysis. This method allows the researchers to gain knowledge through texts based on the participants’ communication about their experiences of a phenomenon, such as SOB [30, 31]. An inductive approach was used to analyse the participants’ answers to the open-ended questions of Exp-BeSoB. Initially, five participants’ answers were analysed by JN, who was guided by the analytic approach, as described by Hsieh and Shannon [30]. Text from each participant was read through to obtain a sense of the whole. All texts were read carefully with an open attitude to the data to capture the qualitative aspects of the experience “being short of breath” and for managing strategies. Meaning units in the text were marked and condensed without losing any content, and given a code, a label of what they said. Reflective notes and analytic ideas were written during this process. Thereafter, the initial analysis, including the coding, reflective notes, and analytical ideas, was jointly reviewed and discussed with ÅR and LB. After that, JN handled the rest of the interviews in the same way and subsequently searched for patterns and relationships. Content of the participants thoughts about SOB, experiences of distress, and the participant’s management of symptoms in daily life, appeared as central and guided the analysis. The codes were grouped based on their differences and similarities, tested and modified into preliminary themes. Further analysis was performed allowing for the discovery and testing of themes emerging from data. Through iterative discussions within the research group, the themes were refined to three final themes that describe the patients’ breathing experiences and the strategies used to manage SOB reflecting a continuum of experiences. Being three researchers involved in the analysis, with differing experiences from care of patients with heart failure, and jointly reflecting on the findings helped ensure that presuppositions and unsupported interpretations were minimized.

### Ethical considerations

This study was ethically approved by the Regional Ethical Review Board of Gothenburg, Sweden (Dnr:265:10) and conforms to the principles of the Declaration of Helsinki [34]. All participants received oral information and signed the written informed consent before participating in the study and none withdrew. Confidentiality was ensured by removing personal identifiers from the participants’ Exp-BeSoB questionnaires and replacing them with codes, a process known as pseudonymization. Participants were referred to by using their unique code number ranging from 1 to 45. This procedure ensured that the participants could not be identified. The code key was securely stored on a password-protected computer and kept separate from the interview data. Only the first author had access to the code key. The coded data was used during the analysis, and the findings were reported in a way that made it impossible to identify the participants involved. Prior to the study, there was no caregiver relationship with any of the participants; however, JN had met some of them (*n* = 8) in her role as a research RN in other clinical trials.

## Results

Five participants were excluded because they had no history of SOB over the last 2 years (*n* = 2), unstable condition (*n* = 1), and unclear genes (*n* = 2). The final sample comprised 45 participants (18 women and 27 men; median age, 74 years) with chronic heart failure. Half of the sample (*n* = 23/45) had heart failure with a reduced ejection fraction, were classified as heart failure functional class III, were diagnosed with heart failure within the last 2 years, and two-third (*n* = 30/45) reported breathing symptoms within the previous week. Comorbidities, such as cardiac artery disease and hypertension, were common. Participant characteristics are described in Table 2.

**Table 2:** Background characteristics in patients with chronic heart failure (*n* = 45)

Age, Year median, (range)	74 (45–90)
Women n (%)	18 (40)
Born in Sweden, n (%)	38 (84)
Married/co-habitant, n (%)	32 (71)
Higher Education (> 12 years), n (%)	7 (16)
Type of heart failure	
HFrEF, n (%)	23 (51)
HFmrEF, n (%)	8 (18)
HFpEF, n (%)	12 (27)
Ejection fraction, %, mean (SD)	39.6 (12,8)
Systolic blood pressure, mm Hg, mean (SD)	128.3 (16.6)
Diastolic blood pressure, mm Hg, mean (SD)	78.3 (11.4)
Heart rate, beats/minute, mean (SD)	65.9 (12.1)
Breathing rate, breaths/min, mean (SD)	16.4 (4.4)
Breathing symptoms within the previous week, n (%)	30 (67)
NYHA class I n (%)	-
NYHA class II n (%)	21 (47)
NYHA class III n (%)	24 (53)
NYHA class IV n (%)	-
Diagnosed heart failure, year range (type value)^1^	< 1–12 (1–2)
< 1 year, n (%)	9 (20)
1–2 years, n (%)	14 (31,1)
2–5 years, n (%)	16 (35,6)
5–12 years, n (%)	6 (13,3)
Comorbidities	
Cardiac artery disease, n (%)	27 (60)
Hypertension, n (%)	22 (49)
Atrial fibrillation, n (%)	16 (36)
Valvular disease, n (%)	18 (40)
Cardiomyopathy, n (%)	10 (22)
Diabetes, n (%)	13 (29)
Renal failure, n (%)	4 (9)
Anemia, n (%)	3 (7)
Obstructive sleep apnoea, n (%)	4 (9)
Chronic obstructive pulmonary disease, n (%)	2 (4)
Depression, n (%)	1 (2)

*HFrEF* heart failure with reduced ejection fraction (<40%), *HFmrEF* heart failure with mid-range or mildly reduced ejection fraction (40-49%), *HFpEF* heart failure with preserved ejection fraction (≥50%), *SD* standard deviation, *NYHA* New York Heart Association

^1^Type value based on year groups

Three themes emerged from the analysis, reflecting a continuum of experiences: *SOB as a life-threatening experience*, *Difficulty breathing slows the body down*,* grabbing one’s focus* and *Breathing as no problem and of no concern* (Table 3.). The degree of symptom distress and applied management strategies differed between and within the themes.

**Table 3:** Overview of the themes

Themes
Shortness of breath as a life-threatening experience
Difficulty breathing slows the body down, grabbing one’s focus
Breathing is not a problem and of no concern

When the participants were asked about their experiences and management of breathing and SOB in a stable phase of heart failure, both current and past experiences of SOB were described. Earlier, fearful experiences of SOB coloured present experiences of SOB. Therefore, as an introduction, these descriptions of acute SOB are presented, before the results of SOB in a stable phase of heart failure. Participants with memories of acute SOB recalled these symptoms as different, frightening, and unmanageable without emergency care. The feelings of suffocation, drowning, panic, and dying, were described as follows:

Yes, when I went in [by ambulance to the hospital], it felt like I was drowning. A long time ago, thought I was going to fall down dead. (P12)

When asked about their present time experiences, 37 (82%) of the participants told of normal breathing and wellness at rest. Additionally, varied personal and multidimensional experiences of SOB, were described. Commonly, SOB in daily life with heart failure was felt to be a lack of air and energy and described as occurring in conjunction with physical exertion and environmental factors, such as stairs and uphill slopes. Symptoms were mostly manageable through resting and breathing techniques that had a calming effect, with a short duration being seen as a healthy sign. Different, frightening, and challenging SOB experiences have also been elucidated.

Many participants had no thoughts about their breathing or SOB in their daily life, while others expressed varied degrees of distress and emotions related to such symptoms. The three themes identified, with different degree of symptom distress are described below. Applied personal management strategies, which differed within and between the themes, are presented integrated in the text.

### Shortness of breath as a life-threatening experience

Shortness of breath could be experienced as a threat to life, even in patients with stable heart failure. Affective descriptions of SOB were characterized as horrible and worrying. Some had frightening experiences of not being able to breathe, which evoked feelings of panic and death. Harder exertion was reported as a trigger, and stress could reinforce and prolong SOB.

SOB as a threat to life, could also be linked to normal physical exertion. Even walking a bit too fast on level ground could be a trigger. The differences between unpleasant SOB, with heart failure, and previous breathlessness connected to exertion, such as running, were described:

*…* Now it’s more like you are not really keeping up, and it’s no pleasant feeling. ….

Oh, my God, how bad [ill] I am, help, I cannot just stand here and die. (P45)

Symptoms were managed by strategies such as waiting for the SOB to subside or standing still with vigorous deep breathing. Sensations of air hunger, being out of breath, and air gasping were described. Caution and avoidance strategies were also applied.

In addition, terrifying episodic bouts of SOB at rest were described. Experiences that previously had required emergency visits to the hospital were described as everything from a life-threatening danger one wanted to avoid to something that was impossible to prevent:

Don’t feel like you can live life to the full … I’m very aware that it [SOB] is there … that it can come. (P35)

Causes of SOB bouts were reported as unclear to the individual, which further contributed to restrictions on normal life. Over time, some learned strategies to manage episodic bouts of SOB, even when these episodes were initially perceived as life-threatening:

… you face it and calm yourself down … the air is for everyone to breath. (P30)

### Difficulty breathing slows the body down, grabbing one’s focus

In this theme, breathing affected the body and mind and was described as difficult, bothersome, and irritating. The participants had difficulties finding words to describe their experiences of being SOB. Descriptions differed, but sensations of “*not getting enough air/oxygen*” were recurring. Breathing was also said to be heavy and/or strained. Some reported a sense of heaviness or tightness in the chest, while others said that their breathing increased and became more rapid. Many described other symptoms such as fatigue or feelings of lack of energy. Overall, SOB experiences associated with physical exertion and sensations of poor fitness. Descriptions of urgent bodily signals to rest or stop and feeling exhausted were common. They struggled with SOB in daily life. Symptoms were managed by strategies such as slowing down and adjusting the pace:

Become annoyed mostly …[thinking] oh my God am I getting short of breath again … it’s hard when you get short of breath, one just struggles and slows down, but I don’t push myself, I slow down pretty quickly. (P10)

Disabling SOB was described in social situations. Symptoms were experienced as emotionally challenging to integrate with one’s self-image:

It (SOB) limits me … It’s bothering me a lot. I survive after all. When I walk from the tram, everyone passes me by, but my God, how slowly I walk. (P 19)

Participants found it tedious and sad to be short of breath and even depressing to think about. One strategy used to manage symptoms was to exercise to improve fitness and participants described that they felt frustrated and even angry when this did not help. Some ignored symptoms and breathing problems and overexerted:

It feels like you’ve run a marathon, a lack of fitness that’s gotten worse … It’s bullocks that it must be like this, bullocks that it [symptoms] doesn’t let go. I don’t feel fear, because I know what it is, that it will pass. … Trying to avoid making myself depressed, but I feel very vulnerable somehow. (P3)

Some participants used strategies such as avoiding activities that triggered symptoms and felt “cut off” from normal life. They sometimes experienced feelings of shame when exhibiting symptoms. Problem avoidance and wishful thinking were also used as symptom management, and envy of others without symptoms, could be discerned:

It puts on the brakes to live like this, it makes me, this that makes you so strained, what you used to be able to do and what everyone else can do, is a holdback, you’re cut off, avoid doing things that are hard, I hesitate to walk up the stairs or bend forward. (P42)

Participants described a complete lack of energy that interfered with their enjoyment of life, making them unable to perform leisure activities:

I get exhausted, I must sit down, become completely devoid of energy and it’s a bit annoying, because it was fun before, when the dog and I could fool around. (P9)

Participants reported that learning in a cardiac rehabilitation program changed their SOB experiences. They learned not to be afraid, as a way to manage the symptoms, and accept heart failure as the cause of exertional SOB. Personality was described as influencing how one felt and managed SOB. Varying severity of symptoms, and episodic SOB from unknown causes, could also be challenging to accept:

It’s strange that you become it [SOB] at different times, with or without effort … tricky … sometimes I can go as far as I want without getting out of breath and sometimes nothing … do not want to accept it, why does it have to be like that? I am a bit stubborn myself; we’re all different as individuals and that certainly affects how you feel. (P17)

### Breathing is not a problem and of no concern

Within this theme, breathing was described as no problem and of no concern. The focus was on physical functioning and managing breathing in daily life. Several participants mentioned that they had previously experienced severe SOB and that they were helped by treatment. Some spoke of new perspectives on life, and had no fear of symptoms or death. Healthy attitudes combined with effective disengaged strategies were also described:

I never think about my breathing it takes care of itself… I become breathless several times a day, it’s almost like a feeling of suffocation… but I don’t care, the beta-blocker takes care of it … Yes [limited] but what’s decisive is the attitude you have, I can’t force my body to do something it can’t do … my capacity today is halved…this has happened, but it must not affect my life. It’s not my brain that’s going to stop me, it’s my body. I’m not afraid of death. (P41)

Applying strategies of not dwelling on symptoms helped some reduce the emotional impact of SOB and was described as *psychological fitness*. SOB could occur very suddenly and even at rest, while others described SOB as normal due to exertion, with a short duration as a health confirmation. Being less fit, with easily triggered SOB and fatigue, could also be attributed to old age or lack of exercise, rather than symptoms of heart failure:

I breathe heavily, but it passes very quickly so I don’t worry … You must stop and exhale … I get tired more easily, but I know what it is … [otherwise] I’ve recovered completely after my by-pass operation … but it’s a bit annoying that you’re so old that you can’t run for the bus anymore. (P1)

Self-taught breathing techniques such as breathing in through the nose and out through the mouth combined with positive visualizations were also strategies applied. Breathing was also managed by altering body posture and shifting the position. Adapting to one’s physical limitations and shifting the focus of one’s attention were also described:

I sit down on a bench if available, and can stretch a bit, if I’m out walking, I can stop and watch the birds until it calms down. (P26)

Participants with easily triggered SOB used strategic planning, problem-solving, and walking aids to manage more demanding activities:

… when I come up the stairs, I have the walker inside the door so I can sit down and rest … when I go uphill … it’s also fortunate to have it. (P4)

Alternating between rest and exertion when performing demanding activities and warming up at a slower pace before increasing the physical load were also symptom management strategies used. Drinking water was a strategy when out of breath due to dry mouth after exercise, as was taking additional diuretics if necessary. Participants told of strategically maintaining the pace and avoiding overexertion that triggered SOB. Others were exercising up to the SOB level as a health and fitness check:

Breathing is no problem … I have control over it … when I’m on the bike and get out of breath … I let go, just roll the bike, then I recover very quickly … signs that my health is quite good … I know what the limit is and settle for that. (P16)

Despite descriptions of SOB causing limitations, some described acceptance, positive emotions, and being grateful that they are alive. Others stated that they knew the reasons for shortness of breath and expressed confidence and trust in their ability to manage it.

## Discussion

The study revealed 45 participants’ subjective experiences of breathing, SOB, and self-developed strategies to manage symptoms of heart failure in daily life. They had different experiences of shortness of breath, and three themes emerged. Some experienced it as a threat to life, in that difficulty breathing slows down the body and grabs the focus, while others thought of breathing as no problem.

In the theme *SOB as a life-threatening experience*, emotional and existential dimensions of breathing symptoms experienced by participants with heart failure were expressed. Descriptions of SOB relating to fear of dying came from participants recalling memories of acute heart failure and needing emergency care. These descriptions are reported as less common for heart failure [14] but were confirmed by participants in the present study, who described breathlessness as “uncontrollable.” This is in line with earlier studies that have described the fear of dying in connection with acute exacerbation in patients with advanced stages of heart failure [13]. In our study, previous life-threatening experiences coloured participants’ experience of SOB, despite being in a stable phase.

The participants also reported strenuous exertion as a trigger for unpleasant and frightening SOB experiences, with feelings of exhaustion and panic, which is not so well known in stable heart failure. Hence, they experienced a sensation of dying triggered by even normal exertion. Rapidly changing health conditions and experiences of a life that is constantly under threat have previously been described in middle-aged individuals with moderate-to-severe chronic heart failure [35]. In the present study, although most reported normal breathing at rest, some participants described fearful experiences of episodic bouts of SOB at rest. Similar findings were also found in people with advanced heart failure [36], as *living in the shadow of fear*. However, the most recent position paper [9] does not cover the needs of patients with heart failure in a stable phase who experience fear related to breathing difficulties.

In contrast, findings within the theme *Difficulty breathing slows the body down*,* grabbing one’s focus* and indicating symptoms of distress but no fear. Participants expressed emotions and how they were limited and bothered by SOB in daily activities. Others avoided activities that made them feel cut off from life. This confirms findings regarding the emotional impact of breathlessness in chronic heart failure [13] and breathing symptoms associated with fatigue and exhaustion. Similar symptoms to those found in our study were also identified in a study that covered different cultural groups [37]. Notable, bendopnea, as described by some participants, is associated with advanced heart failure and increased mortality [38]. Participants who understood that SOB was due to physical exertion and heart failure also experienced unexplained variation from one day to another. They had episodic bouts of SOB at rest but with varied symptoms of distress. This is in line with recent findings that describe this condition as clinically poorly understood [15]. Typically, reactive emotional responses and pacing strategies were applied in this theme. There were also participants who pushed themselves over the limit, resulting in severe symptoms and feelings of sadness. Exercise testing performed in patients with heart failure has indicated that dyspnea and fatigue are cardinal manifestations of heart failure [39]. This suggests that it is important to focus on a broader range of symptoms and self-management strategies. Furthermore, in the present study, content in the participants´ thoughts about SOB, experiences of distress, and management of symptoms in daily life, appeared as central and guided the analysis resulting in the three themes. More resent work on the management of chronic shortness of breath, by the use of the educational tool: Breathing, Thinking, Functioning clinical model [40, 41], support the results, especially in this second theme, of the present study.

Findings within the theme, *Breathing is not a problem and of no concern* sheds light on the possibilities of personal well-being with treatment and learning to manage breathing. Participants related that they felt in the control of SOB and perceived control may promote healthy outcomes. Our study also suggests that those who reported having no thoughts about SOB could have sufficient knowledge about heart failure and self-care or were not aware of SOB as a symptom of heart failure. Previous studies have reported poor symptom recognition in elderly patients suffering from heart failure [18–20]. Also, a meta-analysis showed that patients with heart failure benefit from self-care interventions in terms of less heart failure-related hospitalization [42]. Overall, a bundle of proactive responses and engagement in self-developed management strategies were described in this theme. We cannot assert that those strategies could benefit all but suggest that they have the potential to reduce the unnecessary burden of SOB. This finding emphasizes the importance of clinicians’ understanding of and support for the individual coping processes of patients with heart failure [43].

### The language of breathing and shortness of breath due to heart failure

In medical terminology, shortness of breath is used synonymously with dyspnea and is defined as the “subjective experience of breathing discomfort” [3]. When the participants described their experiences of SOB, they used common, everyday language rather than clinical terms. The risk of mistaking dyspnea as a clinical sign of an underlying disease, manifested as labored breathing, is highlighted [44]. Participants past experiences of breathlessness on exertion differed from the SOB experienced in daily life with heart failure. A variety of everyday expressions were used to describe their SOB, such as “huffing and puffing” and breathing “hard,” indicating laboured breathing that was both physically and emotionally difficult. These differences in medical terminology of dyspnea and patients everyday expressions of breathing difficulties and shortness of breath with heart failure, is important to acknowledge and be aware of, and also to be attentive to in clinical patient encounters.

### Breathing space as a theoretical framework

The proposed concept of *breathing space* [27] may be used as a framework to reflect our findings. The degree of “breathing space,” as related to us by the participants, results from a complex interaction between their management strategies, help-seeking behaviour, and their clinician’s responsiveness to SOB. Most participants were free of symptoms at rest, which can be emphasized as a healthy sign in clinical patient encounters. It was also shown that it was possible to achieve sufficient “breathing space” with applied self-care, including strategies to manage SOB in daily life. Clinicians and researchers have emphasized the importance of recognizing and managing psychological factors [45]. However, those with experiences falling under the first and second themes might benefit from clinicians showing greater responsiveness to SOB as a symptom. Heart failure symptoms, including SOB, are known to cause emotional distress [13, 27, 46, 47], therefore, person-centred care targeting emotional expressions are needed. The third theme indicated that participants might not have been aware that SOB is related to heart failure. This emphasizes the need for education on heart failure and SOB, including strategies for symptom management. Previous research discussed the negative effects of stress on SOB, and how effective symptom management interventions, could improve state of mind in chronic conditions [48, 49]. Although, few studies have focused on supporting patients to manage psychological consequences, signs and symptoms [49]. Experiences and management of breathing and SOB in chronic heart failure, as in other chronic conditions [50, 51] is a somewhat neglected area, where qualitative studies can help fill in gaps in knowledge. The results of this study might potentially be used like a piece in the puzzle for inspiration to develop care that promote appropriate symptom management strategies and well-being in patients with heart failure. Further research is needed.

### Strength and limitations

The present study captures participants’ descriptions of SOB symptoms in daily life despite being considered as having optimized treatment and stable chronic heart failure. A limitation of this study is that the interviews were not recorded, some data might have been lost, even though detailed notes were taken and member checking was used to accurately capture. To ensure meaning equivalence in the translation from Swedish to English, we implemented several strategies. First, all patient quotations were translated by the research team and then reviewed by a professional proofreader fluent in both languages. This process included discussions about cultural nuances, emotional tone, and idiomatic expressions to preserve the original meaning as accurately as possible. When direct translation was not feasible, equivalent expressions were chosen to maintain the intent and context of the original statements. These steps were taken to minimize the risk of losing subtle meanings and to strengthen the methodological rigor of the study. A major strength of this study is that a structured tool with both open-ended questions and comments on descriptors, was used to capture the participants’ symptoms in an efficient and structured way. In order to reduce recall bias of symptoms, both questions on present and past breathing experiences was included in the assessment tool. The open questions and descriptors, in the tool are based on the narrations of patients with stable heart failure [32], which contributes to the usability for use in this patient group.

Methodological *trustworthiness* was ensured by adhering to the established steps of content analysis [30] as outlined in the analysis section, conducting member checking of the participants’ answers during the instrument-assisted interviews and engaging in ongoing author reflexivity. *Credibility* and *confirmability* were strengthened by the authors complementary competencies; JN and LB have specialist nursing experience in heart failure while ÅR and LB bring qualitative research expertise that supported a reflexive analytic process. Trustworthiness was further enhanced by all authors participation in discussions and agreement throughout the analysis and final thematization. Finally the detailed description of the analytic procedures, together with illustrative quotations, provides readers with a solid base for evaluating the *dependability and* overall trustworthiness of the results.

The sample consisted mainly of men (60%) who were followed up at an outpatient clinic at a university hospital. This distribution is considered representative, as coronary artery disease, the most common cause of heart failure, is more prevalent in men than in women. Many participants were also in an early phase of heart failure, which reflects the typical patient population seen in such clinics. At the same time, this focus on relatively early-stage patients may limit the representation of experiences common among older individuals and those with more advanced disease.

The sample included both men and women of older age, with various heart failure diagnoses, common comorbidities, and guideline-directed treatments, yet they remained symptomatic (NYHA class II–III). These characteristics suggest that the findings are relevant for similar clinical contexts in Sweden where patients with stable but symptomatic heart failure are managed in both outpatient and inpatient care settings. The findings may also be applicable to other countries with comparable healthcare structures where dyspnea related to chronic heart failure is a common concern.

However, limitations in *transferability* must be acknowledged, particularly regarding other healthcare systems and cultural contexts. It should also be noted that individuals with the most severe heart failure are not fully represented in the current sample.

## Conclusion

The results help to understand that SOB in chronic heart failure can be experienced in a variety of ways, showing the impact of SOB, and the management of breathing in a stable phase of heart failure under treatment. By asking simple open-ended questions, the patients’ experienced impact of breathing symptoms and their self-developed symptom management strategies for daily life can be captured. This is an important complement to traditional measures of symptoms, such as the NYHA class and rating scales. The open-ended questions of this tool might be implemented during routine visits at out-patient clinics, and also integrated into standard heart failure care or used alongside NYHA and symptom scales. The results also emphasize the importance of clinicians showing responsiveness to patients’ breathing symptoms, also including thoughts about the symptom, emotional and existential dimensions, impact in daily life, and self-management strategies. The result could help clinicians to identify patients who could benefit from individualized education and breathing focused care. Further research is needed on how to best support heart failure patients with SOB to develop self-management strategies, which may also enhance person-centred care. Further work would also be welcome on both the linguistic and emotional aspects of breathing in patients with heart failure.

## Data Availability

All data are collected in Swedish, using an assessment tool: Experiences of Breathing and Shortness of Breath (Exp-BeSoB) in development. The qualitative analysis is performed in Swedish, and the manuscript is translated into English by a native speaking translator. The quotations in the manuscript are shared original data and contributes to the possibility for readers to judge the trustworthiness of the results in the study. Original data (in Swedish), as was used in the present study, may be shared upon request by contacting the corresponding author. Additional data, not included in the manuscript, cannot yet be shared.

## Abbreviations

ATS: American Thoracic Society
Exp-BeSoB: Experiences of breathing and shortness of breath
HFrEF: Heart failure with reduced ejection fraction
HFmrEF: Heart failure with mid-range or mildly reduced ejection fraction
HFpEF: Heart failure with preserved ejection fraction
NYHA: New York Heart Association
SD: Standard deviation
SOB: Shortness of breath
SOB-HF: Shortness of breath in heart failure
